# Anatomical-Based Diagnosis and Filler Injection Techniques: Lips and Philtrum

**DOI:** 10.3390/life15020315

**Published:** 2025-02-18

**Authors:** Gi-Woong Hong, Wonseok Choi, Song-Eun Yoon, Jovian Wan, Kyu-Ho Yi

**Affiliations:** 1 Samskin Plastic Surgery Clinic, Seoul 06577, Republic of Korea; 2 V Plastic Surgery, Daegu, Republic of Korea; 3 Brandnew Aesthetic Surgery Clinic, Seoul, Republic of Korea; 4 Medical Research Inc., Wonju, Republic of Korea; 5 You & I Clinic (Mokdong), Seoul 06001, Republic of Korea

**Keywords:** lip, dermal fillers, hyaluronic acid, rejuvenation, cosmetic techniques

## Abstract

Lip augmentation has become increasingly popular in aesthetic medicine, driven by advancements in dermal filler technologies and injection techniques. This review provides a comprehensive overview of lip anatomy, age-related changes, and current best practices in lip augmentation using dermal fillers. The complex structure of the lips, including multiple layers of skin, muscle, and mucosa, contributes to their unique appearance and function. Age-related changes, such as volume loss, thinning of the vermilion border, and flattening of the philtrum, significantly impact lip aesthetics. Understanding these changes is crucial for developing effective treatment strategies. The review discusses the importance of tailoring treatments to individual patient needs, considering factors such as ethnic variations in lip structure and cultural preferences. It emphasizes the significance of proper filler selection, with hyaluronic acid-based products being the gold standard due to their biocompatibility and reversibility. Injection techniques, including needle and cannula approaches, are described in detail, with a focus on safety and optimal aesthetic outcomes. Anatomical considerations, particularly the vascular supply to the lips, are highlighted as critical for avoiding complications during filler injections. The review also addresses the evolving approach to lip augmentation, which now focuses on restoring natural contours and addressing age-related changes in the perioral region rather than simply increasing volume. Finally, the importance of managing patient expectations and the potential for future advancements in the field are discussed, including the development of more targeted filler products and refined injection techniques.

## 1. Introduction

The aesthetic appearance of the lips plays a crucial role in facial attractiveness and perceived age. Across various cultures and ethnicities, full, well-defined lips with a balanced proportion are generally considered desirable. However, the natural aging process and individual anatomical variations can lead to changes in lip structure and appearance that may be perceived as less attractive. These changes include thinning of the lips, loss of definition in the vermilion border, flattening of the philtrum, and down-turning of the oral commissures [1,2,3,4].

The vermilion border, also known as the vermilion edge, is the distinct line that marks the boundary between the lip’s colored portion and the adjacent normal skin, serving as a critical demarcation for aesthetic procedures. The cupid’s bow refers to the double curve of the upper lip, resembling the bow of Cupid, the Roman god of love; it is a key aesthetic landmark that ensures symmetry and attractiveness in lip enhancement techniques. The philtrum, the vertical groove running from the base of the nose to the upper lip’s border, forms during facial development from the fusion of the nasal and maxillary processes and is essential for understanding both normal facial morphology and congenital anomalies.

In recent years, there has been a significant increase in the demand for minimally invasive procedures to enhance lip aesthetics. This trend is largely driven by advancements in dermal filler technologies and injection techniques, which offer safer and more natural-looking results compared to surgical interventions. Hyaluronic acid-based fillers, in particular, have become the gold standard for lip augmentation due to their biocompatibility, reversibility, and ability to provide subtle yet effective enhancement [5,6,7,8].

Understanding the complex anatomy of the perioral region is crucial for achieving optimal results in lip augmentation procedures. The lips are composed of multiple layers, including skin, muscle, and mucosa, each contributing to their unique structure and function. Furthermore, the vascular supply to the lips, primarily from the superior and inferior labial arteries, requires careful consideration to avoid complications during filler injections. This review aims to provide a comprehensive overview of lip anatomy, age-related changes, and current best practices in lip augmentation techniques using dermal fillers [9,10,11].

## 2. General Considerations

It is often said that one can determine a person’s age by examining the shape of their lips. While cultural differences in the perception of beautiful lips exist across ethnicities and regions, lips that are firm, voluminous, elastic, and have well-defined borders are generally considered attractive. Conversely, thin lips with reduced elasticity that appear inverted and have indistinct borders are typically perceived as less attractive and indicative of aging [12,13,14,15,16,17,18,19,20].

With recent advancements in filler treatments, many individuals are opting for minimally invasive procedures to enhance lip shape and form rather than surgical interventions. There is also a growing trend of people seeking filler treatments to achieve a more youthful appearance of the lips [21,22,23,24].

**Figure 1:**
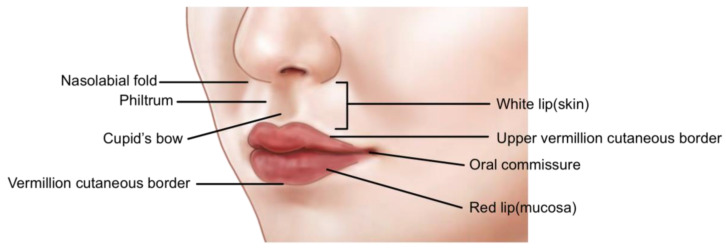
Anatomical structures of the lips.

Between the labial mucosa and the skin lies a slightly raised, line-like structure known as the intermediate zone. The border between this intermediate zone and the skin is referred to as the vermilion–cutaneous border. The bow-shaped indentation in the middle of the upper lip is called the cupid’s bow. The vertically raised skin area between the lips and nose, originating from the lateral peaks of the cupid’s bow, is termed the philtrum. The junction where the upper and lower lips meet is known as the oral commissure (Figure 1) [25,26,27].

**Figure 2:**
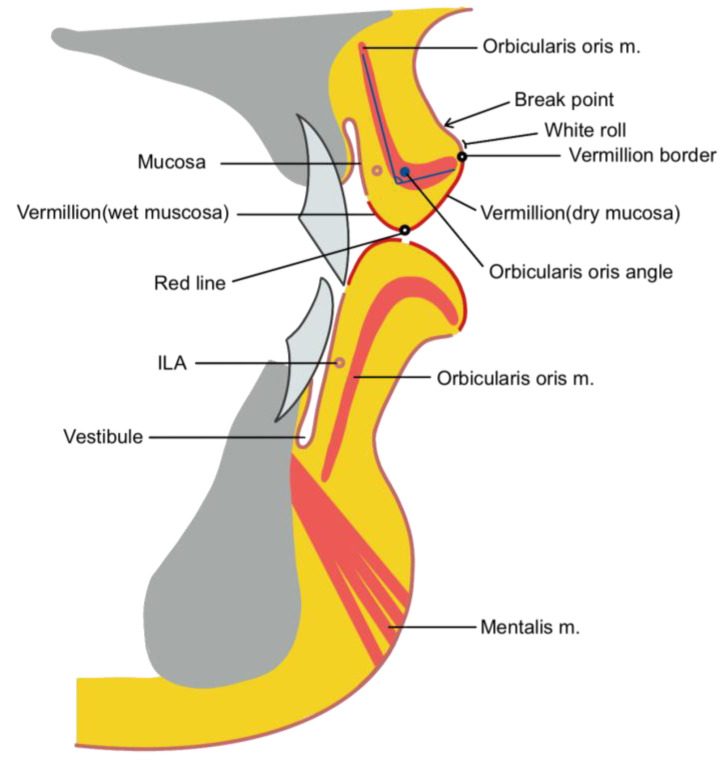
Dry and wet mucosa of the vermilion.

The vermilion region is further divided into an externally exposed dry mucosa and an internal wet mucosa. This demarcation is created by the orbicularis oris angle, formed by the folding of the orbicularis oris muscle within the mucosa. Similar to the supratip break above the protruding nasal tip, a break point can be observed above the slightly bulging area over the vermilion border (Figure 2) [28,29,30,31,32].

**Figure 3:**
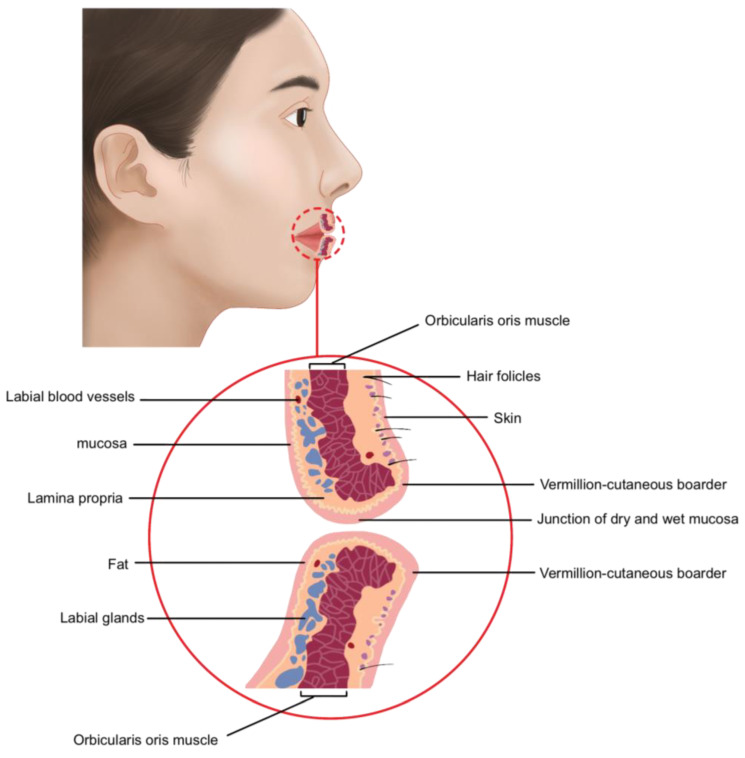
Anatomical layers of the lips.

Examining the lip layers from the exterior, the skin and mucosa of the vermilion border form the outermost layer, with a very thin subcutaneous fat layer beneath. The orbicularis oris muscle lies innermost, with the superior and inferior labial arteries, nerves, and labial glands distributed more deeply. When viewed from the inner wet mucosa side, these vascular and neural structures are located immediately beneath the mucosa. Therefore, caution must be exercised during procedures on the inner lip mucosa to avoid damaging these structures, considering the differences between the outer and inner layers (Figure 3).

When considering the horizontal width of the mouth as defined by the lips, it is generally observed that individuals of African descent have the widest mouths, followed by Caucasians, with those of Asian descent typically having the narrowest. For Asians, an ideal lip width is considered to be approximately 45% of the mandibular width. Regarding lip thickness, individuals of African descent tend to have the thickest lips. Both African and Caucasian populations often prefer slightly thicker upper lips, with a thickness ratio of 4.4:5.5 between the upper and lower lips being favorable in some cases. However, Koreans, who generally have thinner lips among Asian populations, previously aimed for a 1:2 ratio when enhancing the lower lip volume. Currently, a ratio between 1:1.3 and 1:1.7 is considered more appropriate. Therefore, despite racial differences in ideal upper-to-lower lip ratios, a general ratio of 1:1.6 is often considered ideal [4,7,8,11,28,33].

From a frontal view, a slight eversion of the upper lip is desirable, with its lower border positioned 2–3 mm above the upper teeth. In profile, aesthetic appeal is achieved when the upper lip protrudes about 2 mm more than the lower lip.

For the philtrum, the average length in Korean males is 11.5–12 mm, while in females it is 10–10.5 mm. The average central cupid’s bow angle, which relates to philtrum depth, is approximately 130° in males and 135° in females. A moderate philtrum depth is generally necessary for a natural lip appearance and a distinct central cupid’s bow angle. Additionally, the philtral columns and dimples contribute to a natural expression during lip movement. With aging, even without lip inversion, the philtrum’s contours become less defined and flatter, making it appear longer. Enhancing the philtrum’s definition can create a more youthful and softer facial impression by making it appear shorter [3,7,9,10,34,35].

**Figure 4:**
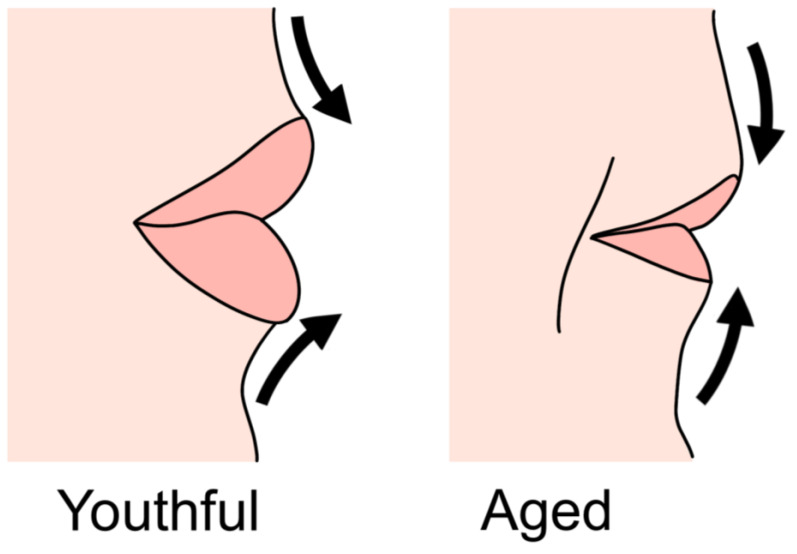
Age-related changes in the lip and philtrum region: thinning and elongation of upper and lower lips, loss of cupid’s bow definition, equalization of lip projection, reduction in vermilion-cutaneous pout, flattening of philtral columns, down-turning of oral commissures, lengthening of the upper white lip, and volume loss in the red lips.

The reasons for seeking lip filler treatments vary. In younger individuals, the primary goals are often to enhance lip volume or to define the lip contours by accentuating the border between the lips and skin. However, as one ages, the shape and volume of the lips change, resulting in flattening and a reduction in vermilion height. The outward-facing volume of both upper and lower lips diminishes, causing them to thin and invert. This leads to a decrease in pouting at the vermilion border and a less defined cupid’s bow. The reduced volume and inward rolling of the lips cause the philtrum to elongate and flatten. Additionally, the oral commissures descend, resulting in a horizontally elongated smile and an increased length of the upper white lip area, which is generally considered less attractive (Figure 4).

Histologically, there is a decrease in collagen and elastic fibers in the mucosal skin, along with an overall reduction in mucosal thickness. Atrophy of the orbicularis oris muscle and blunting of the orbicularis oris angle contribute to the flattening and apparent volume reduction of the lips. While lip volume decrease is considered a natural part of aging, the exact extent and location of this reduction have not been clearly established [1,2,5,36,37,38,39,40].

**Figure 5:**
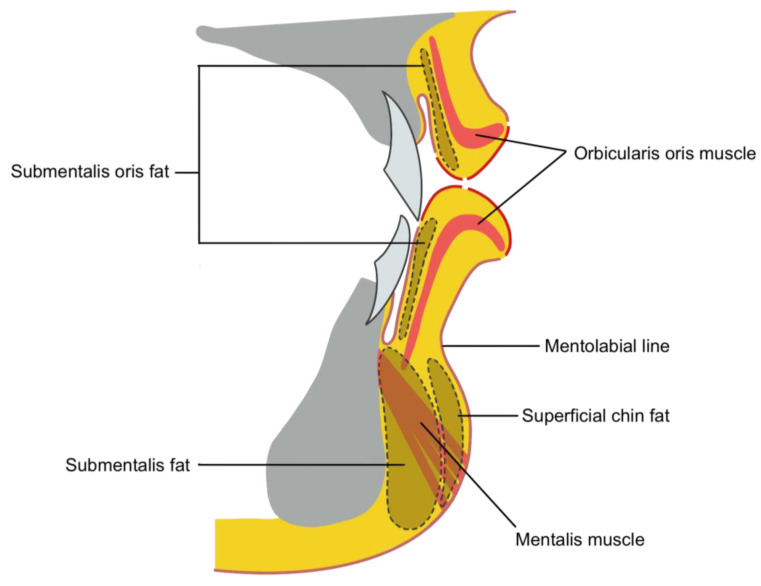
Positioning of the suborbicularis oris fat.

Similar to fat layers in other facial areas, the lip’s adipose tissues, composed of superficial and deep fat, are compartmentalized by the orbicularis oris muscle. Consistent with changes in other facial fat layers, the volume of superficial fat increases with age, while the deep fat layer decreases. Consequently, while muscle and mucosal volume clearly decrease, some argue that the overall lip volume is maintained due to an increase in superficial fat tissue. These studies suggest that filler injections for age-related volume loss should target the suborbicularis oris fat, which corresponds to the deep fat layer between the wet mucosa and muscle of the vermilion (Figure 5).

Regardless of the histological reasons, clinical observations of aged lips show a clear reduction in the dry mucosa of the vermilion, leading to the disappearance of the break point and flattening of the lips, rather than an overall decrease in lip volume. Subsequently, changes in various components of the mucosa, muscle, and adipose tissues result in internal rolling of the lips.

## 3. Anatomical Considerations

To ensure safe procedures, it is crucial to first examine the courses of blood vessels supplying the lips.

**Figure 6:**
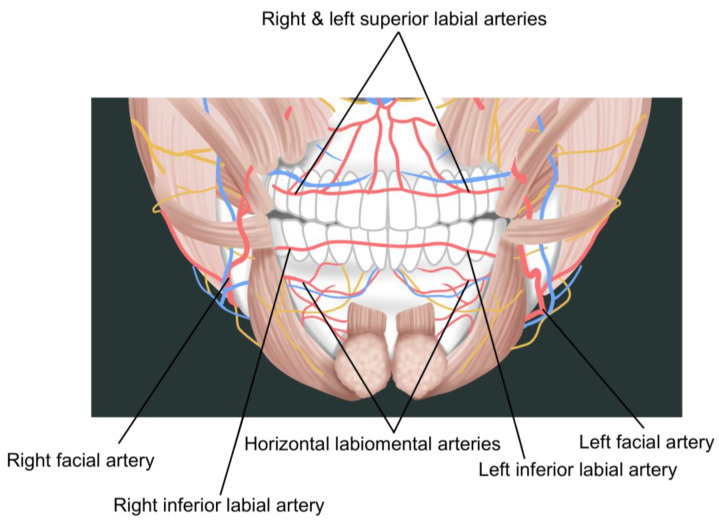
Course of the superior and inferior labial arteries.

The main facial artery, as it courses medially towards the face, gradually approaches the oral commissure and gives off branches toward the lips. The branch supplying the upper lip is called the superior labial artery, with an average diameter of about 1 mm. It typically passes between the mucosa and the orbicularis oris muscle at the same level as the vermilion border, with the left and right branches anastomosing at the midline of the upper lip. The two branches supplying the lower lip are known as the inferior labial artery and the horizontal labiomental artery. While some refer to both collectively as the inferior labial arteries, it is more appropriate to distinguish them due to their distinctly different courses. In approximately 50% of the population, only the horizontal labiomental artery is present, with its ascending branches supplying the entire lower lip. The inferior labial artery fundamentally courses between the inner mucosal surface and the muscle of the lip, indicating that it is positioned closer to the alveolar margin compared to the superior labial artery (Figure 6).

The horizontal labiomental artery, which can be considered the main branch supplying the lower lip area, branches from the facial artery about 15–20 mm below the modiolus. It passes beneath the muscle at a point approximately midway between the upper and lower borders of the depressor anguli oris muscle, typically following the labiomental crease between the lower lip and chin. As mentioned earlier, in about half of the population, a branch can be observed emerging slightly above the origin of the labiomental artery, coursing along the vermilion border of the lower lip. This vessel is termed the inferior labial artery, and in some cases, anastomoses can be observed between these two vessels. The superior labial artery branches from the medial aspect of the winding portion of the facial artery near the corner of the mouth and progresses along the vermilion border of the upper lip [6,41].

**Figure 7:**
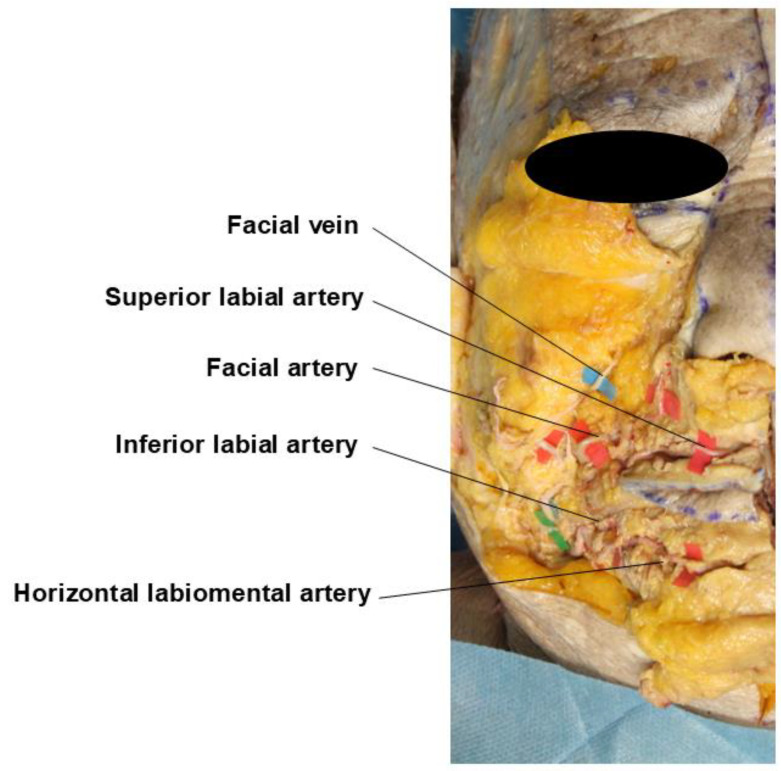
Topography of major perioral vessels.

Previously, it was believed that the labiomental artery was the main inferior labial artery, typically coursing along the labiomental crease with some branches ascending towards the lower lip. This led to the assumption that filler injections at the vermilion border carried a relatively lower risk of vascular injury in the lower lip compared to the upper lip. However, just as the superior labial artery branches near the corner of the mouth and courses along the vermilion border, current opinions suggest that a separate inferior labial artery exists, following the vermilion border of the lower lip. Consequently, deep subdermal injections at the vermilion border of both upper and lower lips should be considered high risk for vascular injury. Therefore, it is safer to position the entry point for needles or cannulas at a distance from the corner of the mouth, where the upper and lower vessels begin to branch and course (Figure 7) [2,6,40].

The left–right branching pattern of the vessels supplying the lips is symmetrical in only about half of the population, with asymmetry being common. This is particularly true for the superior and inferior labial arteries and the horizontal labiomental artery, where development on one side often corresponds with underdevelopment on the opposite side. Thus, it should not be assumed that vessels follow identical paths on both sides during bilateral procedures.

## 4. Site-Specific Treatment Methods

When injecting fillers into the lips, using excessively firm fillers can result in lumpiness and patient discomfort due to foreign body sensation. Conversely, using overly soft fillers and over-inflating can lead to a swollen appearance without the desired firmness. Therefore, it is advisable to use fillers of moderate firmness with good viscosity rather than elasticity, as the lip area is constantly moving and should recover well after deformation.

Before the procedure, it is essential to assess lip asymmetry and determine the overall required volume. Generally, 0.5–1 mL of filler is used for each lip, with a maximum of 1.5 mL per lip even in cases requiring larger volumes. Excessive filler amounts tend to thicken the lips rather than appropriately rotating them outward, resulting in an undesirable swollen appearance. It is beneficial to explain this to patients undergoing the procedure.

There are generally two main areas for lip filler injection: along the vermilion border on the skin side and into the mucosal side of the lip. Injecting on the skin side allows for lip enhancement with smaller amounts of filler and is particularly beneficial for patients lacking a prominent break point at the lip border. However, this approach may not be suitable for patients with a visible break point, as it might disrupt their existing lip contours. Mucosal side injections, on the other hand, can maximize vermilion eversion while preserving the existing break point, making it more suitable for such patients. However, this method may require more filler to achieve the desired volume enhancement compared to skin-side injections.

**Figure 8:**
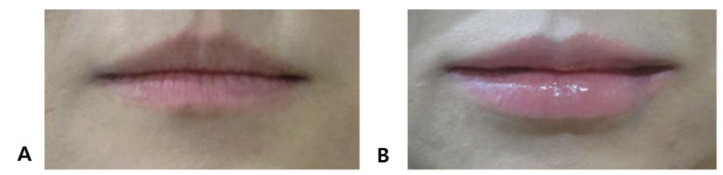
Lip filler injection: before (**A**) and after (**B**) treatment comparison.

The decision to emphasize the small central tubercle in the middle of the upper lip should be made in consultation with the patient. Generally, East Asian patients often desire emphasis on the central tubercle, while most Western patients prefer not to accentuate this area. For the lower lip, it is common among East Asian patients to create two tubercles on either side of the center, more pronounced than the central area. However, the current trend among younger patients favors an overall plump appearance without tubercles for both upper and lower lips, so the decision should be based on the patient’s preference. When emphasizing tubercles on the upper and lower lips, a small amount of additional filler can be used to enhance these specific areas. Figure 8 shows a female patient in her 20s with a slightly enhanced central tubercle on the upper lip and more pronounced lateral tubercles on the lower lip, creating a so-called dumbbell shape.

**Figure 9:**
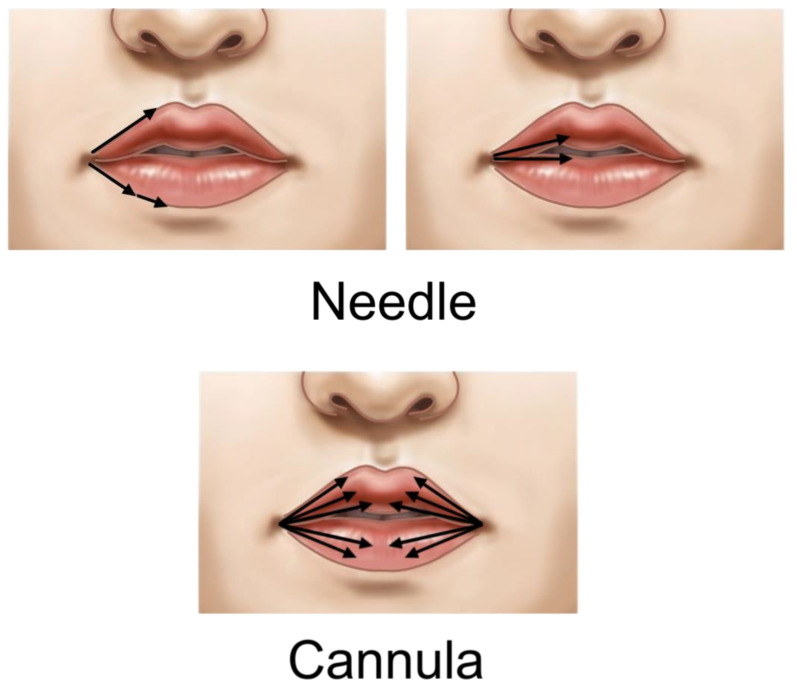
Lip injection techniques. Needle technique: Entry point 2–3 mm medial to oral commissures; vermilion–cutaneous border and cupid’s bow treated with linear threading and serial puncture; lip mucosa treated with retrograde fanning in the submucosal layer. Cannula technique: Entry point in skin lateral to cheilion; vermilion–cutaneous border and cupid’s bow treated with linear threading; lip mucosa treated with retrograde fanning in the submucosal layer.

The filler injection sequence typically begins by defining the vermilion border to enhance the contours of the lips and cupid’s bow, with injections made into the dermal and subdermal layers. After enhancing the contours, if additional volume is desired or if the lips appear thin and inverted, filler can be injected submucosally at the dry–wet mucosa junction. This helps increase volume in this area, resulting in greater eversion and enlargement of the entire lip (Figure 9). To avoid lump formation, injections should not be made too superficially beneath the dermis or mucosa. However, caution is needed to avoid deep injections beneath the muscle from the skin side or deep submucosal injections from the inner mucosal side, as these can damage branches of the superior and inferior labial arteries, potentially leading to severe bleeding and associated complications.

The procedure should be performed with the patient in a seated position, typically starting with the lower lip, which is usually larger, and then adjusting the upper lip’s shape and size accordingly. Both needles and cannulas can be used for filler injection. Needles are preferable for creating defined contours or adding volume to specific areas for more precise shaping. Cannulas, being less prone to causing bleeding, are generally safer for overall volume enhancement, starting from the outer lip and progressing to the deeper mucosal areas.

When using a needle, starting 2–3 mm inside the oral commissure and injecting towards the center can prevent bulging at the cheilion (corner of the mouth). However, in cases where the lower lip is very thin and lacks volume, causing the upper lip corner to cover the lower part and giving the appearance of a downturned mouth corner, enhancing the volume at the corner can make the lower lip end visible, improving the appearance of a downturned corner and creating a smile-like shape. Conversely, if the mouth corner position is good but the outer part of the upper lip is thin, enhancing this area can create a slight downward bulge of the upper lip inside the mouth corner, giving the effect of an upturned corner. When using a needle, the vermilion border and cupid’s bow contours can be enhanced using linear threading or serial puncture techniques, while lip volume can be increased using the retrograde fanning technique (Figure 9).

For cannula use, the skin is punctured slightly outside the mouth corner and entered using linear threading and retrograde fanning techniques to enhance contours and volume. As with needles, care should be taken to avoid excessive bulging at the mouth corners. While it is possible to inject filler into both upper and lower lips through a single entry point using the retrograde injection technique, separate entry points for upper and lower lips, spaced about 1 cm apart, can be used if necessary. The basic injection method is similar to that of needles, but when enhancing lip volume, it is preferable to inject the filler more towards the inside of the lip to minimize visible marks (Figure 9).

**Figure 10:**
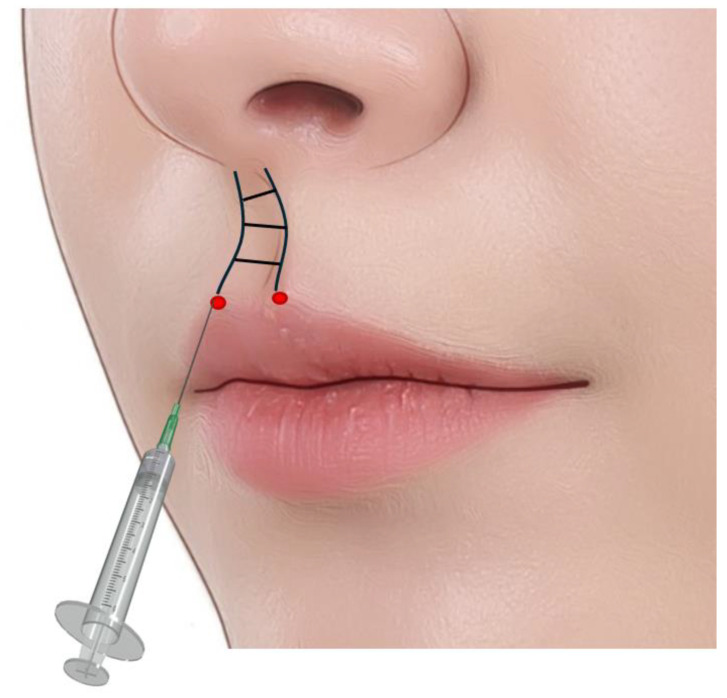
Philtrum injection technique: Entry point at vermilion line towards philtrum; retrograde linear threading or tiny serial punctures in the subdermal layer including deep dermis.

While enhancing lip contours and volume, the philtrum can be accentuated by injecting filler from the vermilion line towards the philtrum if needed. The philtrum area can also be injected using a needle or cannula into the dermal and subdermal layers using the retrograde linear threading technique or tiny and serial puncture injection techniques to define its borders and contours (Figure 10).

**Figure 11:**
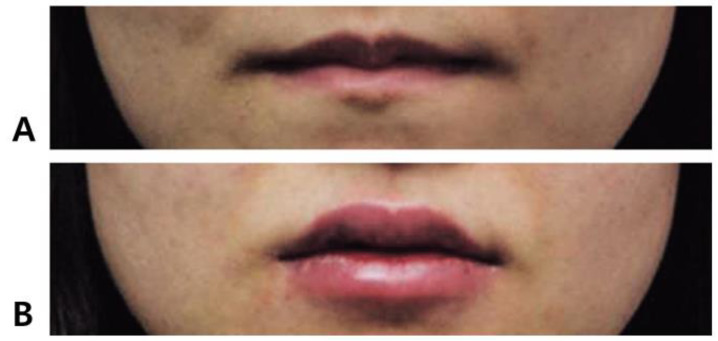
Filler injection for thin lips before (**A**) and after (**B**) treatment comparison.

Figure 11 shows a female patient in her 30s whose lips lacked volume and pouting at the vermilion border rather than being thin. Due to the habit of tightly closing the mouth, characteristic of Korean oral structure, her lips appeared thinner than they actually were by rolling inward. The condition was corrected using a moderately firm, viscous filler, injecting 0.6 mL into the upper lip and 0.7 mL into the lower lip.

In this case, the patient desired a fuller appearance for the upper lip, so the volume ratio between the upper and lower lips was planned to appear as 1:1.3. First, injections were made along the vermilion border to define the lip and cupid’s bow contours while sufficiently enhancing the pouting of the previously inward-rolled lips, creating an effect of ample volume. Next, a small amount of filler was injected submucosally at the dry–wet mucosa junction on the inside of the lips to enhance volume in this area, creating an eversion effect for the lips. This approach aimed to improve overall lip volume and facial impression around the mouth.

Immediately after the procedure, it is advisable to apply an ice pack until there are no signs of bleeding to minimize bruising and swelling at the injection sites. Massage of the treated area may also be necessary to ensure even distribution of the injected filler for a smooth appearance. Patients should be reassured that even properly placed filler gel inside the lips may initially feel lumpy, and there may be a foreign body sensation when the tongue touches the lips, but this will feel natural over time. It should also be explained that temporary swelling is a common symptom following lip procedures.

Furthermore, patients should be informed that about 10–20% of the initial volume will subside as swelling reduces within 1–2 weeks. Managing patient expectations is crucial, and it is advisable to explain in advance that achieving natural results may require a two-stage approach, with treatments spaced 2–4 weeks apart, rather than a single session [2,6,40].

## 5. Complications and Management

Complication management of lip fillers using hyaluronic acid (HA) requires a systematic approach due to the variety of potential adverse events that may arise. Common complications include skin discoloration, edema, nodules, infections, and vascular compromise, each with distinct clinical presentations and treatment strategies. Skin discoloration, such as bruising or the Tyndall effect, can often be managed conservatively with topical treatments like vitamin K ointment or, in persistent cases, laser therapy. Edema, ranging from transient post-procedural swelling to delayed hypersensitivity reactions, may require antihistamines, corticosteroids, or hyaluronidase depending on its etiology. Inflammatory and non-inflammatory nodules can develop due to improper injection technique, filler migration, or immune responses, and treatment varies from massage and aspiration to hyaluronidase administration and, in severe cases, intralesional corticosteroids or 5-FU injections [42,43,44].

Infections, though rare, pose significant risks and may present as cellulitis, abscess formation, or biofilm-related delayed reactions. Management typically involves systemic antibiotics, drainage when necessary, and the removal of filler using hyaluronidase. Vascular compromise, one of the most serious complications, requires immediate recognition and aggressive intervention. Early signs, such as blanching or livedo reticularis, necessitate high-dose hyaluronidase injections, warm compresses, and possibly anticoagulant therapy [21].

## 6. Discussion

The approach to lip augmentation has evolved significantly with our growing understanding of lip anatomy and the aging process. While traditional techniques often focus solely on increasing volume, modern approaches emphasize the importance of restoring natural contours and addressing age-related changes in the perioral region. This includes not only enhancing the vermilion but also supporting the white roll, defining the cupid’s bow, and addressing the philtrum and oral commissures [2,36].

Lip augmentation aesthetics demonstrate significant ethnic variation, with traditional Caucasian ideals (1:1.6 upper-to-lower lip ratio) not necessarily applying across different populations. Asian measurements show notable diversity: Chinese women have a 1:1.25 upper-to-lower lip ratio, Korean women range from 1:1.11 to 1:1.25, and Asian women generally have fuller lips than Caucasian women, though men show less ethnic variation. Hispanic populations, particularly Mexican Americans, tend to have more protrusive lips (2–3 mm upper lip, 0.6–2.4 mm lower lip) compared to European Americans, though detailed measurements are limited in the literature. Cultural preferences also vary significantly: Asian populations generally prefer smaller lips (with Koreans favoring slightly fuller lips than Japanese), while Latin American populations prefer larger lips compared to US and European standards. This research underscores the importance of considering individual ethnic backgrounds and preferences rather than applying a one-size-fits-all approach to lip augmentation [7].

A study by Lorenzo et al. [45] presented a comprehensive analysis of lip augmentation assessment methods, focusing on advanced 2D and 3D imaging techniques. The study evaluated a commercial lip plumper in 50 healthy women over 30 days, using stereophotogrammetry and various measurement tools to quantitatively assess changes in lip volume, shape, and health parameters. The results showed significant improvements, including a 6.5% increase in volume, a 15% improvement in the Upper Lip Index, and a 30.4% enhancement in hydration levels. The researchers employed sophisticated imaging systems (VECTRA H2 and VISIA) along with multiple measurement techniques to provide a thorough analysis of lip enhancement outcomes. While the study demonstrated the effectiveness of these measurement methods for evaluating lip augmentation results, it acknowledged limitations such as the short follow-up period and limited demographic range. This research represents an important step forward in establishing quantitative standards for assessing lip enhancement procedures, whether through cosmetic or aesthetic approaches.

A study by Noury Adel [36] introduced an innovative “Inverted Mercedes Benz” technique for lip filler injections, characterized by using three strategic entry points—two at the Glogau–Klein points of the upper lip and one at the midline of the lower lip, forming an inverted triangle pattern. The technique employs a 22 G 50 mm microcannula to inject hyaluronic acid filler in both retrograde and aliquot fashion within the superficial muscular plane, offering several advantages over traditional needle-based methods. In a study on 10 female patients aged 22–29, the approach demonstrated enhanced safety with a reduced risk of vascular occlusion, better artistic control for defining the cupid’s bow and lip tubercles, and minimal complications compared to conventional “Russian lips” techniques. While 70% of patients experienced mild temporary swelling and bruising (mainly at entry points), there were no serious adverse events, and all patients reported high satisfaction. The technique specifically avoids filler spread into the ergotrid area, which can create an undesirable “mustache appearance”, though the author recommended further studies with larger sample sizes and suggested that a shorter cannula might provide better injection.

Kempa et al.’s [46] study used eye-tracking technology and surveys to analyze how 101 participants (52 females, 49 males) perceived lip aesthetics in relation to facial proportions. Rather than finding a single universal “golden ratio”, the research revealed that different ratios were considered most attractive for various facial features. Key findings included that the most attractive vertical lip position had a 1:2 ratio (distance between lips and chin being double the distance between lips and nose), while the optimal lip width to bigonial distance ratio was 1:2.5. For upper lip vermilion thickness, males found 1:3 most attractive, while females preferred 1:2, and both genders found a 1:4 ratio most attractive for lower lip vermilion thickness. Importantly, the study found that what is considered most attractive is not necessarily what is perceived as most masculine or feminine—for instance, a prominent male chin might be rated as more masculine but less attractive. The research also validated the “internal representation of beauty theory”, as participants identified unattractive features more quickly than attractive ones. These findings provide valuable quantitative guidelines for aesthetic practitioners while emphasizing the need to consider individual preferences and gender-specific characteristics in facial aesthetics.

A study by Werschler et al. [47] described the development and validation of the Allergan Lip Fullness Scale (LFS), a standardized tool for evaluating lip augmentation outcomes. The research progressed from an initial 4-point scale (iLFS) to a revised 5-point scale (LFS) that better accommodates diverse facial presentations, particularly fuller lips common among African Americans. The validation process involved 8 physicians rating 55 live subjects for the iLFS and 21 clinicians evaluating 144 3D images for the LFS across two sessions separated by at least 14 days. Both scales demonstrated substantial to almost perfect inter- and intra-rater reliability among physicians, with the LFS showing inter-rater agreement of ≥0.69 and strong intra-rater agreement. The study established that a 1-point change in the LFS represents a clinically meaningful difference in lip fullness. Key features of the LFS include separate assessments for overall, upper, and lower lips and the inclusion of diverse ethnic representations in the scale examples. The study concluded that the LFS is a reliable tool for physicians to classify lip fullness and can be valuable for discussing treatments, setting goals, and documenting outcomes in clinical practice, though it is not recommended for patient self-assessment.

One of the key considerations in lip augmentation is the selection of appropriate filler products and injection techniques. Hyaluronic acid fillers with varying rheological properties are now available, allowing practitioners to tailor their approach based on the specific area being treated and the desired outcome. For instance, softer fillers are often preferred for subtle volume enhancement in the body of the lip, while firmer products may be used for defining borders or addressing deep perioral lines [35,41].

The potential for vascular complications remains a significant concern in lip augmentation procedures. Recent anatomical studies have highlighted the variability in the course of the labial arteries, challenging previous assumptions about safe injection zones. This underscores the importance of a thorough understanding of vascular anatomy and the use of techniques that minimize the risk of intravascular injection, such as the use of blunt cannulas or specific injection depths and angles [4,11].

Patient satisfaction and natural-looking results are paramount in lip augmentation. Achieving these goals requires not only technical skill but also an artistic eye and an understanding of individual facial proportions and cultural aesthetic preferences. Moreover, managing patient expectations and providing clear communication about potential outcomes and limitations of the procedure are crucial aspects of successful lip augmentation. As the field continues to advance, future research may focus on developing more targeted filler products, refining injection techniques, and exploring combination therapies to address the multifaceted aspects of perioral rejuvenation.

## Data Availability

The data are available by contacting to corresponding author.
